# Exploring the role of commercial actors and structures in contributing to sexual violence in live music venues

**DOI:** 10.1093/heapro/daag148

**Published:** 2026-10-06

**Authors:** Gabriel Caluzzi, Felicity Wardle, Sarah Clifford, Ruth Canty, Cassandra J C Wright

**Affiliations:** Menzies School of Health Research, Charles Darwin University, Red 9 Casuarina Campus, Univ Drive North, Casuarina NT 0811, Australia; Centre for Alcohol Policy Research, School of Psychology and Public Health, La Trobe University, Kingsbury Dr, Bundoora VIC 3083, Australia; Menzies School of Health Research, Charles Darwin University, Red 9 Casuarina Campus, Univ Drive North, Casuarina NT 0811, Australia; Menzies School of Health Research, Charles Darwin University, Red 9 Casuarina Campus, Univ Drive North, Casuarina NT 0811, Australia; Drug Policy Modelling Program, Social Policy Research Centre, University of New South Wales, Kensington NSW 2052, Australia; College of Medicine and Public Health, Flinders University, Cnr University Drive North & University Drive West, Brinkin NT 0810, Australia; Menzies School of Health Research, Charles Darwin University, Red 9 Casuarina Campus, Univ Drive North, Casuarina NT 0811, Australia; Centre for Alcohol Policy Research, School of Psychology and Public Health, La Trobe University, Kingsbury Dr, Bundoora VIC 3083, Australia; Burnet Institute, 85 Commercial Rd, Melbourne VIC 3004, Australia

**Keywords:** sexual violence, live music venues, alcohol, commercial determinants of health, alcohol, qualitative methods

## Abstract

Sexual violence is a significant public health issue, and increasing attention has focused on sexual violence in live music venues, particularly the social and environmental features that contribute to it. While research on the commercial determinants of health has expanded, it has not been applied to sexual violence. This study examines how commercial factors, including commercial practices and structures, contribute to sexual violence in live music venues. We draw on interviews with 25 staff and patrons from live music venues in the Northern Territory, Australia, conducted in late 2024. Participants were asked about barriers and facilitators to preventing sexual violence. We analysed interviews thematically first using an inductive approach, then deductively applying a commercial determinants of health framework to examine how commercial actors and structures might facilitate sexual violence. Commercial actors were seen to engage in market practices (e.g. exceeding capacity, overserving alcohol, understaffing) and non-market practices (e.g. symbolic engagement with prevention initiatives) that contributed to sexual violence. These practices occurred within systems that prioritized profit and produced a casualized workforce often ill-equipped to respond to sexual violence. Sexual violence was further reinforced by gendered power relations in venues, a reliance on prevention through self-regulation, and neoliberal norms that framed sexual violence as an issue of individual responsibility. Addressing commercial drivers through regulation offers actionable policy levers—although economic structures may constrain these efforts. We suggest greater attention to commercial determinants is both theoretically and politically valuable in sexual violence research across live music settings and other environments.

Contribution to Health Promotion

- An explicit focus on commercial actors and structures has not previously been applied to sexual violence, including in commercial settings such as live music venues.
- Drawing on interviews with venue patrons and staff, findings show that corporate decisions can create conditions that enable sexual violence.
- These decisions are embedded within commercial structures that prioritize profit and reinforce norms that enable sexual violence.
- Identifies opportunities to improve safety through stronger regulation, while acknowledging that broader commercial incentives can undermine these efforts.
- Introduces a commercial determinants framework to sexual violence that could inform future policy and research.

## Introduction

Sexual violence is a significant public health, gender equality, and human rights issue across the world (World Health Organization/London School of Hygiene and Tropical Medicine 2010). It encompasses a continuum of behaviours, ranging from unwanted sexual contact such as groping or leering to attempted rape and rape (Kavanaugh 2013). In Australia, 14% (2.8 million) of adults have experienced sexual violence since the age of 15, including more than one in five women (22%) and one in 16 men (6.1%) (Australian Bureau of Statistics 2023). Rates of sexual violence are also particularly high among non-binary and LGBTQ+ people (Hindes *et al*. 2026). Impacts of sexual violence include immediate and long-term physical, psychological, social, and economic consequences (Centres for Disease Control and Prevention 2022). The drivers of sexual violence are multifaceted and include both structural factors such as gender inequalities and social norms (Our Watch 2021), as well as situational factors such as alcohol consumption (Abbey 2011, Caluzzi *et al*. 2025a). While sexual violence occurs across diverse settings, growing attention has focused on live music venues, including pubs, bars, and clubs, as well as music festivals (Fileborn 2016, Fileborn *et al*. 2020, Hill *et al*. 2020, Hill and Megson 2020, Button *et al*. 2024, Price and Driscoll 2025). In this article, we look at how commercial factors contribute to sexual violence in live music venues.

### Live music venues and sexual violence

We use the term ‘live music venues’ to refer to quasi-public hospitality venues where various genres of live music are played, such as bars, pubs, and nightclubs. These venues are often hybrid sites of consumption (e.g. food, drink, events) that provide important social, cultural, and economic benefits. They contribute to local economies and support the music industry, offer sites for relaxation and leisure, and can be settings where people seek romantic or sexual partners (Fileborn 2016). However, these environments can also function as hotspots for sexual violence, particularly for young people (Hill *et al*. 2020, Price and Driscoll 2025). While it is difficult to determine prevalence due to underreporting, several studies provide insights. One study from the US surveying 1091 live music attendees found that 61% of attendees had experienced sexual harassment or assault in their lifetime, including 82% of women (Price and Driscoll 2025). In another study of sexual harm in Australian nightlife settings (including live music venues), researchers surveyed 232 patrons and found that 57% of women and 32% of men reported experiencing nightlife-related sexual harm in the past 3 months (Button *et al*. 2025c). A further study of 126 predominantly female attendees of live music venues in Australia found that 60% of respondents felt unsafe in live music venues (Baker 2023). In addition to patrons, sexual violence is also likely to be prevalent among hospitality workers in live music venues (Coffey *et al*. 2023).

There are several factors linked to sexual violence in live music venues. Environmental features such as darkness, crowding, loudness, and reduced visibility can enable perpetration and limit opportunities for intervention (Button *et al*. 2025a). Alcohol and other drug consumption can facilitate perpetration, both by increasing the risk of perpetration among men predisposed to sexual aggression (Abbey 2011) and by undermining victim-survivors’ ability to consent and seek help (Caluzzi *et al*. 2025b). Hyper-sexualized atmospheres that emphasize overt displays of sexuality, masculinity, and femininity can devalue and objectify women, while normalizing unwelcome sexual behaviours (Fileborn 2016, Sanchez *et al*. 2019). This means that actions that appear less severe on the continuum of sexual violence, such as unwanted touching, groping, coercion, and verbal harassment, are common and pervasive in the context of nightlife settings (Kavanaugh 2013). For example, high rates of sexual violence mean many women ‘expect’ harassment on a night out attending a venue (Thompson and Cracco 2008, Fileborn 2016), while inconsistent or inadequate venue responses act as barriers to reporting (Hill *et al*. 2020, Button *et al*. 2025b, Price and Driscoll 2025).

Interventions in these settings typically operate at the individual or venue level (Button *et al*. 2024). Individual approaches often promote self-monitoring and reduced alcohol consumption, positioning safety as a matter of personal responsibility. Such strategies risk reinforcing the gendered dynamics of sexual violence by constructing (usually female) victims as accountable for preventing their own victimization (Fileborn 2016, Price and Driscoll 2025). Indeed, prevention efforts frequently prioritize women’s self-protection over addressing perpetration (Quigg and Bigland 2020), thereby reproducing victim-blaming narratives. Venue-level initiatives include staff training to respond to sexual violence, bystander-awareness campaigns, and having designated safe spaces (Button *et al*. 2024). While promising, their implementation is also constrained by resource pressures and high staff turnover (Hill and Megson 2020).

### Situating sexual violence within commercial structures

In public health, there has been an increasing recognition of the role of commercial actors (e.g. corporations, businesses) and systems (e.g. consumer capitalism) in producing negative health outcomes and inequalities—known as the Commercial Determinants of Health (CDOH) (Kickbusch *et al*. 2016, de Lacy-Vawdon and Livingstone 2020, Gilmore *et al*. 2023). The CDOH are defined as ‘the systems, practices, and pathways through which commercial actors drive health and equity’ (Gilmore *et al*. 2023, p. 1195). Profit motives, commercial objectives, and the promotion of unhealthy products and choices can conflict with public health goals (de Lacy-Vawdon and Livingstone 2020). For example, traditional and social media corporations can expose young people to marketing of health-harming products (alcohol, tobacco) in ways that avoid regulation (Pitt *et al*. 2024). The growth of transnational corporations focused on financial profit can similarly undermine the health and working conditions of unskilled workers (Friel *et al*. 2024).

While the focus has largely been on health-harming, profit-driven industries known to drive rates of non-communicable diseases such as processed food, tobacco, and alcohol (Stuckler *et al*. 2012), there is growing recognition that commercial entities can influence social, political and economic systems to harm health in more obfuscated ways (Gilmore *et al*. 2023). For example, corporate practices can influence policy, including undermining public health regulations, challenging scientific evidence, promoting unhealthy product use, and laying blame for harms on particular populations (Ulucanlar *et al*. 2023). Indeed, contemporary capitalism has meant that areas of public policy and industries operate with varying degrees of commercial influence (Friel *et al*. 2023). CDOH scholarship therefore emphasizes looking at the wider social and economic structures that contribute to inequity and health harm, such as neoliberal market economies, corporate structures, globalization, corporate political activities, and harmful production practices (de Lacy-Vawdon and Livingstone 2020). Harm, therefore, arises not only from deliberate corporate strategies but also from structural conditions that normalize and prioritize commercial imperatives.

While sexual violence is increasingly recognized as a structural problem linked to gender inequities (Armstrong *et al*. 2018, Our Watch 2021, United Nations Office of the High Commissioner on Human Rights 2026), it often remains framed in terms of proximal risk factors and individual behaviours (e.g. Centres for Disease Control and Prevention 2022). Such framings can be harmful because they divert attention from the broader systems that enable sexual violence. Instead, sexual violence is more usefully understood as a harm shaped by social, commercial, and political systems, including the norms, regulations, and policies that structure people’s environments and behaviours (van Schalkwyk *et al*. 2022). Consistent with contemporary CDOH definitions (Gilmore *et al*. 2023), we conceptualize sexual violence within commercial systems, including corporate practices, incentives, and governance arrangements that create or reinforce conditions that enable sexual violence. Indeed, CDOH frameworks and structural theories of sexual violence share a common emphasis on power and how unequal (and gendered) power relations are embedded within institutions and social systems (Armstrong *et al*. 2018, Wood *et al*. 2022, McCarthy *et al*. 2023). As Hill (2024) notes, commercial determinants often (re)produce structural gender inequities; companies promote harmful gender stereotypes and constructions of masculinity/femininity, while pushing the sale of products like alcohol that exacerbate gender-based violence. Thus, CDOH frameworks offer a useful lens through which to understand the commercial contributors to gender inequities, including those that enable and reproduce sexual violence.

A focus on commercial elements is particularly useful for understanding sexual violence in live music venues for several reasons. First, contemporary CDOH definitions highlight that commercial entities can have not just negative effects but also neutral and positive effects (Gilmore *et al*. 2023). Indeed, there is a range of heterogeneous commercial entities with varied goals (Lacy-Nichols *et al*. 2023). This is well reflected in live music venues, which involve multiple commercial entities (e.g. venue owners, music promoters, alcohol suppliers) and provide social, cultural, and economic benefits for local industries and communities beyond our focus on sexual violence. Second, there is growing acknowledgement that CDOH frameworks should be applied beyond narrow domains of health science (Hagenaars *et al*. 2024) to better understand harms that fall outside of traditional disease models (van Schalkwyk *et al*. 2022). Indeed, there is increasing recognition that addressing commercial determinants can play a role in reducing sexual violence (Hill and Salter 2024, Hill 2024). For instance, alcohol regulation has been identified as a key mechanism for reducing sexual violence (Lippy and DeGue 2016), as has gambling regulation for intimate partner violence (Dowling *et al*. 2016). Third, previous research has highlighted how barriers to effective venue-based prevention are often rooted in commercial imperatives, such as time and financial costs to train staff (Hill and Megson 2020, Button *et al*. 2024). Finally, CDOH frameworks have started being applied to violence, with an acknowledgement that, like other public health issues, the role of commercial actors and structures is often obscured (Bellis *et al*. 2024). To our knowledge, no study has applied a CDOH perspective to sexual violence.

In this article, we consider both downstream (e.g. practices directly driving harms) and upstream (systems indirectly enabling harm) commercial determinants of sexual violence in live music venues. We draw on interviews with staff and patrons of live music venues in the Northern Territory (NT) in Australia. The NT has a geographically dispersed population, and many live music venues in the NT operate as hybrid spaces, serving food and beverages and hosting both live and recorded music (e.g. DJs) at different times of the day. Darwin and Alice Springs are the two main population hubs with populations of 139 902 and 36 471, respectively (Australian Bureau of Statistics 2021a, 2021b). Compared to other Australian states and territories, the NT also has the highest per capita rates of sexual violence (198 per 100 000) (Australian Bureau of Statistics 2024). Against this backdrop, we aimed to examine how commercial factors contribute to sexual violence in live music venues.

## Methods

This study was part of a larger mixed-methods evaluation of a programme (the All Good Project) designed to increase safety for patrons, performers, and staff in live music venues across the NT (Wardle *et al*. 2025). The component we report on involved qualitative interviews with 25 staff and self-identified regular patrons of live music venues in the NT to understand their perceptions of the barriers and facilitators to preventing sexual violence. Ethical approval was obtained from the Menzies School of Health Research Human Research Ethics Committee (2022-4267).

Participants were recruited through several live music venues in the NT that had agreed to take part in the All Good Project. To recruit staff, information was emailed to venue managers with contact details of the research team. Research team members then attended the venues in person to interview consenting staff and patrons. To further recruit patrons, posters were distributed at venues with details of the study. Snowballing recruitment was also undertaken, and the study was promoted via an online social media group. All participants were provided with a Participant Information Statement and had an opportunity to ask questions or discuss any concerns before deciding whether to participate. Verbal informed consent was recorded at the commencement of the interviews. Our previous research found that, when discussing potentially sensitive topics around alcohol and other drug use and sexual harassment, participants prefer verbal consent procedures over written consent.

A total of 25 participants took part in the qualitative interviews, including 14 patrons and 11 staff. Participants were from Darwin (14 participants, 56%) and Alice Springs/remote areas (11 participants, 44%). Overall, 17 participants were female, and eight were male. Within the staff group, three participants were venue managers. We note three participants identified as live music performers in addition to their role as a patron or venue staff, and many staff also reflected on their experiences as patrons of live music venues. Moreover, most staff had worked in and were reflecting on experiences working across multiple venues, often comparing and contrasting the one they were working in currently to others they had worked in or frequented. To that end, findings reflected experiences across a range of venues.

Semi-structured interviews were conducted between August and November 2024. Interviews lasted 25–40 minutes and were conducted either face-to-face, over the phone, or via video call by F.W., S.C., and C.W. The interview guides (see Supplementary files S1 and S2) explored staff and patron experiences or observations of sexual violence and harm, their view of the programme, and barriers and enablers to change at venues. In this study we examined the latter. We also chose not to explicitly use the term ‘sexual violence’ in the interviews. Rather, we described it as behaviour that made participants feel unsafe/uncomfortable, with examples to avoid ambiguity. These included being creepy, leering, following, rubbing, groping, catcalling, and harassing, as well as other forms of violence and discrimination. Interviews were recorded, transcribed using the MS Word transcription function, checked for errors, cleaned to improve grammar and anonymize names and places, and imported into NVivo (v12, QSR) for further analysis.

### Data analysis

Coding and analysis involved an iterative and recursive process that included both inductive and deductive analysis. First, coding was conducted inductively by G.C. and F.W. G.C. and F.W. independently read and open-coded several transcripts, discussed thematic concepts deemed important and what codes might contribute to these, and synthesized their codes. For example, G.C. and F.W. decided that reflections on commercial actors and practices were a central concept and used a specific code to reflect this—*Financial or commercial barriers and enablers to safety*. Other relevant codes for this study included *Regulations* and *Venue safety practices*. F.W. then coded the remaining data accordingly, refining, adding, or amending codes as needed.

Second, data analysis followed an inductive thematic approach. Specifically, a reflexive approach to thematic analysis supported by best-practice guidelines (Braun and Clarke 2021, 2024). Reflexive thematic analysis is a theoretically flexible method for ordering data that can draw on both inductive and deductive analysis (Braun and Clarke 2021). We found it to be a useful way of organically coding data and iteratively refining themes. Taking a reflexive thematic approach guided by our research question (how do commercial factors contribute to sexual violence in live music venues?), G.C. organized coded data into inductive themes such as *Profit models producing environmental risks* and *Commercial spaces reinforcing power relations*.

Finally, after discussions between the co-authors, we decided our preliminary analysis suggested our inductive themes could be more comprehensively explained through a CDOH framework. In this way, our analysis was shaped by a critical realist perspective, which seeks to understand how subjective individual experiences are shaped by external political, organizational, economic, and social structures (Vincent and O'Mahoney 2018, Kersey *et al*. 2023). Consistent with a theoretically informed deductive orientation in reflexive thematic analysis (Braun and Clarke 2024), we subsequently used a CDOH framework as an interpretive lens through which to re-read and make sense of our inductive themes. After reviewing several CDOH frameworks, we applied an adapted framework proposed by Lee *et al*. (2022). This framework examines commercial determinants as both decisions and practices of commercial actors (such as strategies to increase corporate profit) and how these operate according to structural forces that shape commercial logics (such as neoliberalism) (see Table 1). While the adapted framework did not inform the research question or interview guide and was not decided on *a priori*, applying it deductively allowed us to test the utility of a pre-existing CDOH framework. It also offered a useful lens through which to interpret findings in a way that emphasized structural, rather than individual, approaches to sexual violence. We found the framework aligned closely with our inductive themes and enabled us to distinguish between proximal and distal commercial factors shaping sexual violence. G.C. then led the application of the deductive framework to the pre-existing inductive themes. The framework not only captured the pre-existing themes but also encouraged us to return to the data to examine latent codes that we missed during the initial coding process—particularly around *stratification* and *norms*.

**Table 1:** Conceptual framework for the study of commercial determinants of health (adapted from Lee *et al*. 2022).

Actor		
Components	Definition	Example
Market strategies	Actions businesses take to maximize profits	Product design, advertising, and promotion
Non-market strategies	Actions and strategies businesses use to increase their power and influence and decrease uncertainty	Corporate social responsibility activities, lobbying
Structure		
Political and economic systems	Patterned network of relationships between institutions, groups, and individuals	Systems of production, welfare, and labour markets
Stratification	Society’s categorization of its people into groups based on social factors	Unequal distribution of power among different social groups
Governance	Rules and processes by which a society steers itself towards goals	Tax codes and legal codes
Norms	Standards or patterns of behaviour, based on prevailing beliefs and values, considered normal or expected	Individualization, privatization

Importantly, the data and analysis were shaped by our disciplinary backgrounds (sociology, psychology, and public health) and our knowledge of sexual violence and the live music sector, including both experiential and professional expertise. Our analysis was also informed by an agenda of social justice and gender equity and an interest in understanding systems-level contributors to sexual violence that go beyond the individual. This perspective enabled us to interpret participants’ accounts not only as descriptions of individual experiences but as indicators of the commercial processes shaping the conditions in which sexual violence occurs.

## Results

We present our results below according to the different CDOH components in the Lee *et al*. (2022) framework—*market* and *non-market strategies*, *political and economic systems*, *stratification*, *governance,* and *norms.* We present quotes below using participant numbers, staff/patron status, and sex.

### Actor—market strategies

Participants spoke about how commercial actors who owned live music venues—often people who did not physically work in the venue themselves—made financial decisions that contributed to unsafe environments. For example, participants described venues increasing their patron capacity to unsafe levels of crowding, overserving of alcohol, and being understaffed. Here, participants attributed unsafe environments to businesses and owners wanting to maximize financial gains.

*I think there's other venues that just go ‘that's outside of the business model that we're operating, which is selling alcohol, selling entertainment to people. What happens with safety is more of an individualistic level responsibility, and we only respond if it becomes a problem.’ And that sounds a bit cynical, but I think there are a lot of venues in the NT that operate with that mindset, and they're run by business people that don’t think about social and safety, sexual violence and physical violence aspect of it. They just treat it like a business model. (Patron #6, male)*

Increased capacity limits were mentioned by several staff members and patrons. For example, one staff member described how their venue had recently doubled their capacity, meaning there were ‘more intoxicated people’ and ‘obviously more incidents compared to before’. Some specifically noted how increased capacity numbers were tied to business owners wanting to make more profits selling alcohol. Indeed, selling alcohol, and selling to already intoxicated customers, was described as a business directive of some venues that contributed to unsafe environments.

*You have to sort of go along with what the venue wants, you know, it's sort of up to the venue. Some venues will be a bit strict on that and say like, we don't want really drunk people here. And other venues, they just want people's money and they're not really keen on you cutting people off from alcohol. So I guess just generally having people, you know, really drunk or whatever, that's just in itself unsafe. (Staff #5, male)*

Understaffing of key roles such as security due to profit margins was also a common issue raised by participants. They described how this contributed to both an inability of staff to respond to or notice sexual violence, while also undermining the capacity to prevent violence in a venue.

*I know a lot of places now yeah like I said they don't have many security guards or they don't have many staff working because they're really trying to push down on making money. (Patron #12, female)*

One staff member also noted that a focus on patrons as consumers also meant staff safety was often overlooked.

*For staff safety it [zero tolerance policy] should be at the forefront. Like for a lot of venues the consumer is at the forefront and I get that that's like the most important part of having a business […] but like we don't deal with these problems. (Staff #4, female)*

These reflect revenue-maximization strategies that actors, usually business owners, employed and which produced unsafe environments that enabled sexual violence.

### Actor—non-market strategies

Other non-market strategies described by participants involved activities intended to demonstrate social responsibility and minimize reputational risk but which often had limited impact on preventing sexual violence in practice. For example, while the sexual violence prevention initiative evaluated as part of this study received positive feedback from staff, management, and patrons (Wardle *et al*. 2025), how much it was embedded in venue practice reflected different degrees of commitment. For some venues, their uptake of the programme was reflective of a broader commitment to addressing sexual violence and included addressing environmental risks, developing relationships with security staff, and strong leadership from management (‘it does sort of change the culture’—Staff #5, male). However, several patrons and staff noted that voluntary participation in sexual violence prevention initiatives might allow other venues’ management to participate in largely symbolic ways aimed at maintaining public appearances (i.e. corporate social responsibility) and observed that efforts to address sexual violence could be limited to that without other meaningful actions taken.

*I think it happens often in a lot of spaces where they'll say that they have this policy or this approach and you don't really see it coming through properly. And sometimes it feels like it can just be a tokenistic thing of, hey, we say we support this thing. (Patron #1, female)*

Participants suggested that when venues lacked genuine commitment to preventing sexual violence, these initiatives could displace responsibility for preventing sexual violence away from venues—leaving patrons responsible for managing their own safety.

*How is there any embodying these or manifestation of these [safety] values that you're putting on the walls? Yeah, and the community having to take it into their own hands, spread the word that that person is unsafe, but you're like, wait there should be a larger structural way of dealing with this. There should be a police report, but also the venue should be doing more at that level. (Patron #6, male)*

They later added:

*I could imagine that a venue would say, yeah, we do all these things. We do all these things [sexual violence response initiatives]. But if that clashes with their business model or something, will they actually do it? (Patron #6, male)*

These non-market strategies revolved around voluntary participation in sexual violence prevention initiatives. While initiatives were broadly supported and participants viewed reduction of sexual violence in venues as important, greater commitment from venues to meaningfully take action to reduce risk factors through operational and environmental changes was seen as critical.

### Structure—political and economic systems

Participants both explicitly described and implicitly alluded to broader political and economic systems that undermined efforts to prevent sexual violence in live music venues, particularly neoliberal attitudes towards profit and labour. Some of these pressures stemmed from profit maximization and alcohol-centred business models embedded in nightlife economies, which encouraged actors to enable high-risk environments (as described above). Many barriers to prevention were also linked to labour conditions within the hospitality sector, including precarity, understaffing, and casualized employment.

High staff turnover made it difficult to train workers in sexual violence prevention and policies. As a result, responsibility for safety was often transferred to workers with limited training or support. As one manager noted:

*To be honest like running a small business we only have limited time to train people. Like, I could not spend a week training everyone and all the single policies we have. (Staff #6, female)*

Precarious employment conditions among frontline workers also created barriers to staff reporting incidents to managers. Participants often discussed this in the context of staff experiencing sexual harassment themselves, alongside informal expectations to prioritize customer satisfaction over their own safety. For example, one staff member noted:

*…drunk guys or whatever, just giving you, the bar staff or other, mostly women in the venue a hard time or, maybe making them feel uncomfortable. I guess that would be pretty common. (Staff #5, male)*

And later added:

*People you know aren't inclined to speak up for themselves or anyone else against the manager's wishes because it's so casualized, like you literally have to look for a new job. So I think that's a big problem. (Staff #5, male)*

An industry culture of understaffing and overwork, shaped by pressures to maximize profitability and minimize labour costs, also contributed to these issues. Some staff described working in conditions where limited staffing, high workloads, and fast-paced commercial operational models reduced their capacity to adequately monitor or respond to incidents of sexual violence.

*Even if the venues are really proactive on trying to pick up unwanted behaviour there's probably like too much going on from […] a workload perspective […] they just got like a lot of other things to worry about. Like that could easily slip under their radar. (Patron #10, male)*

Distance between venue owners and frontline staff could contribute to low prioritization of safety. One participant referred to venues run by a stereotypical ‘faceless kind of businessman’ with no ‘direct connections’ to the venue, resulting in a stronger focus on profit than safety. In this way, venues were described as operating within commercial systems that prioritized revenue extraction, while being embedded in labour markets that directly constrained safety capacity.

### Structure—stratification

Stratification in live music venues was described primarily through the reinforcement of gendered power relations. While sexualized behaviour was often normalized and commercialized, participants spoke of how these environments tended to privilege men and masculinity over women’s safety—particularly when sexuality was linked to a venue’s commercial reputation (i.e. having a ‘hookup culture’). This reflects how sexual violence is embedded within gendered power relations that are amplified in commercial settings like live music venues. For example, while the gendered nature of sexual violence was often implicit in discussions with participants, some patrons noted that sexual violence in venues was ‘almost always’ perpetrated by men against women—often in ways that enabled men to enact power.

*And it's almost always, and I can't even think of a time when it isn't so, it feels stupid that I have to like, say that. But it's always men hassling women, usually younger women who are smaller and they can feel like they have power over them. (Patron #1, female)*

Sometimes these gendered dynamics formed part of the commercial identity or reputation of particular venues. For example, venues known for a ‘hookup’ culture were seen as environments where women were more vulnerable, described by one participant as a ‘sexual hunting ground.’ Others noted how business practices, such as operating over capacity, interacted with gendered power dynamics to produce conditions that heightened women’s vulnerability in live music venues.

*Let's say, like a place like [venue] or somewhere that feels over capacity, even, like with the privilege I have, I still do feel quite claustrophobic at times and overwhelmed. I feel unsafe sometimes in those sorts of settings. I can't imagine how a woman feels like or someone who's more vulnerable, I can imagine in a setting like that would be a nightmare like because it's so hectic. And it's so loud, and there's just so many people around, it’s very overwhelming. (Staff #3, male)*

While the stratification of sexual violence varies across a range of social factors (Armstrong *et al*. 2018), gender was the primary axis of sexual violence stratification within live music venues according to participants. In particular, some venues’ commercial reputation promoted highly sexualized environments and unsafe conditions (such as exceeding capacity) that reinforced gendered power dynamics and undermined women’s safety.

### Structure—governance

The issue of governance and regulation of sexual violence prevention and alcohol was raised—sometimes explicitly or implicitly. Taking part in sexual violence prevention programmes was voluntary and part of a self-regulatory system of governance. There were no mandated requirements from venues, and many staff described feeling unequipped to deal with it prior to receiving the All Good training. For example, participants talked about how there were no guidelines around managing or preventing sexual violence in live music venues (‘There’s no kind of like handbook or manual’—Patron #1) and how addressing unwelcome sexual behaviour often went unacknowledged in venue governance. ‘It’s not like, oh, if you do this certain sort of behaviour then you have to leave […] I don't think they [management] talk much about, like other sort of behaviours, like harassment or you know, things like that’ (Staff #5, male).

Others described how venues regularly overserved alcohol despite liquor regulations, suggesting that the revenue generated from alcohol sales may outweigh the cost of potential liquor infringements. For example, staff who sell or serve alcohol anywhere in Australia must undertake Responsible Service of Alcohol training, which covers alcohol sales laws and the refusal of service to intoxicated patrons. Venues must also comply with liquor legislation and licence conditions intended to prevent service to intoxicated patrons and those on the Banned Drinker Register (an NT policy that prohibits the purchase of alcohol by individuals identified as causing alcohol-related harm to themselves or others) to minimize alcohol-related harm. However, some participants felt that these obligations were rarely enforced and routinely breached. This undermines the effectiveness of alcohol certifications as a mechanism for limiting excessive consumption—a known driver of sexual violence in live music venues.

*I think there's a lot of problems with Responsible Service of Alcohol as well, yeah. Constantly we see some of the same places, [pub] and [bar/pub], receive infringements from I suppose the Liquor Commissioner for either serving people who are on the Banned Drinker Register alcohol, or not scanning licences, or just way over serving people alcohol with no safety nets in place or no mechanisms to slow things down over the counter in bars as well. (Patron #6, male)*

While most participants emphasized venues and venue managers as the primary actors responsible for preventing and responding to sexual violence, a handful also highlighted the importance of broader governance structures, such as government policy and legislation, in establishing more consistent measures.

*There's also a responsibility on a government level to make sure that they set up or, that they establish like good policy and legislation to try and prevent that as much as possible, yeah. I think the like the kind of management is a like a multi-faceted one […] I feel like those kind of policies should be a uniform across multiple venues so you should expect the same response from whatever venue you go to. But then also like those policies could also be driven by well let's take guidelines or policy legislation as well. (Patron #10, male)*

While participants did not always explicitly discuss the governance of sexual violence, their responses frequently pointed to a regulatory absence in how venues prevented and responded to it, as well as a lax approach to liquor licensing and overserving in some venues.

### Structure—norms

Prevailing neoliberal logics about individualization and personal responsibility played into sexual violence prevention and response systems in venues in several ways. It meant that safety was often framed as a matter of individual and bystander responsibility and rarely one of overarching regulations. It also meant there was an acceptance of harassment and ‘rowdiness’, rather than a zero-tolerance approach to sexual violence. These contributed to creating and normalizing live music venues as risky environments for sexual violence.

Some participants described the high frequency of sexual violence creating an expectation within venues that patrons would take responsibility for their own safety and refrain from reporting unwanted behaviour, highlighting how profit-oriented environments can contribute to the tacit acceptance of sexual violence.

*If whoever was managing the venue at that night had every person that was unwantingly touched, or you know what I mean, like come up to them, it would just be…I feel like it would just be too much like. Like they wouldn't be able to do anything, necessarily to every single person. (Patron #4, female)*

Others described a degree of tolerance or acceptance of sexual violence occurring in venues, framing it as an inevitable feature of live music venues.

*Look in a perfect world that shouldn't happen. But I don't know how realistic it is to cut that kind of behaviour out from venues 100%. I still think they should [prevent it] but there'll still always be an element of those kind of events happening. (Patron #10, male)*

While the participants we spoke to did not directly link these norms and expectations to commercial drivers, neoliberal frameworks that underpin commercial systems often prioritized individual responsibility and self-regulation. In this context, these dynamics appeared to contribute to commercial norms that normalized and legitimized live music venues as environments of sexual violence.

## Discussion

Sexual violence in live music venues has become an area of growing concern among researchers (Fileborn 2016, Fileborn *et al*. 2020, Hill *et al*. 2020, Hill and Megson 2020, Button *et al*. 2024, Price and Driscoll 2025). However, to our knowledge, existing work has not examined the role of commercial drivers. This is important because nightlife spaces have increasingly become sites for corporate power (Chatterton and Hollands 2003), which goes against the sense of community and care that patrons might consider central to safety in live music venues (Fileborn 2016). A focus on commercial drivers also enables us to go beyond aetiological models that focus on risk factors and individual choices to better understand how policy landscapes and social structures facilitate harm (van Schalkwyk *et al*. 2022). This includes commercial contributions to gender inequity through harmful norms, stereotypes, and the promotion of products known to contribute to gender-based violence (McCarthy *et al*. 2023, Hill 2024). While analyses of the CDOH and critical theories of sexual violence share many similarities, particularly their emphasis on how embedded power relations shape experiences of harm and inequality, to our knowledge, a CDOH framework has not yet been applied to sexual violence. Given suggestions by others that CDOH should be applied beyond health sciences (Hagenaars *et al*. 2024), we propose that applying it to sexual violence offers both theoretical and practical value.

Here, we highlighted how both commercial actors and broader commercial structures can produce environments that enable or prevent sexual violence. Participants described alcohol-centred profit models, venues pushing capacity limits beyond safe levels, and a focus on symbolic participation in prevention programmes as examples of commercial decisions and practices undertaken by venue operators. At a structural level, the casualization of the hospitality labour force, the reinforcement of gendered power relations in music venues, reliance on self-regulatory approaches to prevent sexual violence, emphasis on individual responsibility, and the tacit acceptance of sexual violence also shaped these environments.

While there is growing recognition that many drivers of sexual violence are rooted in upstream inequalities (Our Watch 2021), a CDOH lens directs us to examine how corporate entities and commercial systems contribute to gender inequities (McCarthy *et al*. 2023) and shift blame onto individuals (Gilmore *et al*. 2023). These logics of personal responsibility were reflected in our data, where people anticipated inadequate venue responses. This echoes the broader research base on individual-level sexual violence interventions that highlights how, while they can be effective, such interventions can also individualize the problem and contribute to victim blaming when implemented in isolation (Fileborn 2016, Price and Driscoll 2025). The varying degrees of venue engagement with sexual violence prevention we encountered further point to limited problem recognition among venue operators and a broader lack of accountability for harms occurring within these commercial environments.

Indeed, many participants described the highly gendered nature of sexual violence and a tacit acceptance of it in live music venues. This included sexual violence among staff. While nightlife spaces like live music venues are known to actively commodify gender and sexuality as part of their appeal to patrons (Chatterton and Hollands 2003), this commodification also contributes to the broader normalization of sexual violence in spaces where women consume alcohol (Caluzzi *et al*. 2025b), highlighting how CDOH reinforce gender inequities in such settings. While structural norms and gender inequalities are difficult to shift, policy and regulation responses offer an alternative avenue for action. Notably, incentives encouraging overselling alcohol and exceeding venue capacity were levers we identified where there seemed to be an inadequate regulatory response. Addressing alcohol overserving and unsafe environments, alongside policies to prevent and deter sexual violence, can form part of a broader suite of interventions targeting nightlife settings such as live music venues (Quigg and Bigland 2020).

Our analysis also highlights how sexual violence is mediated by relationships between venue managers, corporate owners, and broader social and economic structures that enable self-regulation. This was reflected in voluntary, at times symbolic, commitments to sexual violence prevention, distance between business owners and venues that diluted accountability, and the dismissing of infringement notices as part of business operations. Recognizing that sexual violence in commercial spaces such as live music venues and other nightlife settings is tied to profit imperatives means that, while management can do a lot to create safe spaces, financial imperatives undermine this. This may strengthen the case for regulatory interventions. Such regulations would need to be both consequential to enforce compliance and to reshape norms around responding to sexual violence. There are already examples of this happening in Australia. New South Wales and Victoria have both integrated sexual harassment and assault into their Responsible Service of Alcohol Training (NSW Government 2025, Victorian Government 2025), while the NT has integrated content directly from the All Good Project (Support Act 2025). South Australia also recently announced mandatory industry-wide training for hospitality venues (Government of South Australia 2025). These regulatory shifts reflect the growing evidence linking commercial industries like alcohol to gender-based violence and an acknowledgement of the need for policy responses. Similar evidence and advocacy in live music settings could inform policy action elsewhere.

Finally, there are commercial incentives for venue operators to adopt sexual violence prevention initiatives. Commercial actors are conscious of the importance of maintaining their social licence to do business and prioritize building and maintaining relationships with the public and civil society (Cullerton and Patay 2024). Accountability for maintaining a safe environment can be framed as commercially beneficial, and our data highlighted patron cynicism towards venues perceived as primarily profit-driven or only taking symbolic action against sexual violence. Conversely, well-defined safety initiatives may function as a commercial asset, helping to build a positive reputation that attracts patrons and supports staff retention (Music Victoria 2026). However, it is important that such efforts contribute to meaningful, substantive approaches to safety—and preferably are upheld through external oversight.

There are several limitations to our study. While we suggest that findings are likely to be applicable elsewhere, they should be understood within the specific context of the NT. Staff may have also been unwilling to discuss facilitators of sexual violence in their workplace, particularly given the often-precarious nature of their employment. Moreover, while we gave specific examples to avoid ambiguity, we acknowledge that our focus on behaviour that made people feel unsafe or uncomfortable may have shaped participant responses—particularly given the normalization of sexual violence in live music venues. For example, participants may not have interpreted less overt behaviours (e.g. leering or sexual comments) as sexual violence, whereas different wording (e.g. ‘unwanted sexual attention’) might have elicited different experiences. The decision to apply a CDOH framework was also made during the analysis rather than guiding data collection. This means specific questions on commercial drivers were not included—instead, our analysis was guided by our application of a commercial determinants lens to what were often latent codes. Our findings also reflect the themes most salient within each framework category but are likely not exhaustive. However, the fact that commercial drivers were recognized in the data despite not being an *a priori* focus highlights their underlying importance in staff and patron perspectives. More research on sexual violence using a commercial perspective is needed, particularly within commodified spaces and known ‘hotspots’ for sexual violence like live music venues.

Research on sexual violence in live music venues is growing, and our analysis suggests these harms are strongly shaped by commercial drivers. While commercial drivers will operate differently across contexts, we suggest that attending to them is both theoretically valuable and politically useful in sexual violence research across live music settings and other environments. Thus, we echo calls for CDOH frameworks to be applied beyond the health sciences, including to social scientists looking to understand societal harms (Hagenaars *et al*. 2024). A focus on commercial determinants allows us to think about the production of harm beyond proximal risk factors, while shining a light on the diffuse ways that commercial structures contribute to gender inequities and harms. Indeed, a CDOH perspective that holds both commercial actors and the broader structural conditions that contribute to sexual violence accountable can provide insights into policy levers and responses.

## Supplementary Material

daag148_Supplementary_Data

## Data Availability

The participants of this study did not give written consent for their data to be shared publicly, so due to the sensitive nature of the research, supporting data is not available.
