# Supplementary material for: Exploring the role of commercial actors and structures in contributing to sexual violence in live music venues
Source: Health Promot Int. 2026 Oct 6;41(5):daag148. doi: 10.1093/heapro/daag148 (PMC13639480; doi:10.1093/heapro/daag148)
Supplement: Supplementary file 1 [file daag148_supplementary_data.zip › Supplementary file 1 - Interview guide (staff).docx]

**Supplementary File 1: Interview guide (staff)**

Hi I am [name] from Menzies.

We are doing research with Music NT to help pubs and clubs respond better when there is trouble at venues.

We can have a 10-20 min chat to hear your thoughts.

At the end of the interview, we have a $40 woollies voucher to give you.

Its anonymous and confidential.

We can do the interview now or take your details and do it by phone or zoom at another time that works for you.

1. Informed consent/plain language statement process.

- *Your manager is supportive of us being here interviewing you, but even still, everything you say is confidential, not identifiable to you and private from your manager and venue. We put everyone’s responses together and then summarise it.*

2. Information regarding participant

- *Role at venue*
- *How long they’ve been working at [venue]*
- *Age*
- *Gender and pronouns*

We are interested in understanding safety in NT music venues

3. So what I would like to begin with is with exploring what it is like in [venue name]

- *Do you think it is a safe venue for people to party in?*
- *Do you think it is safe for all people?*

4. Can you tell me about the venue culture at [venue name]?

-	What is it like to work here?

-	What do you think the reputation of the venue is for patrons?

5. Can you tell me about a time at [venue name] when you experienced or noticed behaviour that made you or someone else feel uncomfortable?

- *like someone being creepy, leering, following you around, rubbing up against you or groping you, catcalling you*
- *like someone harassing you,*
- *or aggressive or violent behaviour like pushing, slapping, punching, aggressive looks*
- *like being yelled at or someone being verbally abusive or derogatory or discriminatory,*
- *or like being discriminated against based on race, gender, disability, religion, or some other personal characteristic, experiencing racism*
- *or even more violent behaviour like sexual or physical assault*
- *What did you or other staff do?*
- *What did other people at [venue name] do?*
- *How often does that sort of thing happen?*
- *Do you hear about it happening to other people?*
- *Have there been other times for you?*
- *What do you see your role as when you notice these types of harms?*

*5.	Can you tell me about a time at [venue name] when you experienced or noticed behaviour that made you or someone else feel unsafe?*

*-	What did the staff do?*

*-	What did other people at [venue name] do?*

*-	How often does that sort of thing happen?*

*-	Do you hear about it happening to other people?*

*-	Have there been other times for you?*

*-	What do you see your role as when you notice these types of harms?*

6. How would you expect staff at the venue to respond?

*Do you notice how performers respond? Or security? Or bar staff?*

7. Do you see other people stepping in to keep others safe?

*Could be friends or strangers*

8. All Good Project recall

- *Have you noticed any posters, materials or other messaging in this or other venues that relate to discrimination, violence or anything we’ve discussed so far?*
- *What have you seen/where did you see it?*
- *Have you seen this type of messaging in other places in the NT or elsewhere?*
- *Do you remember what you thought when you first saw it?*

This venue has signed up to a project called the All Good Project, which trains staff to respond to harassment and discrimination.

- *Is there anything else you can recall that reflects this venue’s involvement?*
- *What do you think about venues being involved in these kinds of projects?*

9. Have you done any of the All Good training?

-	How was it for you?

-	Was there anything that impacts your day to day work at [venue name] now?

*-*	Have there been any changes in the venue culture since the AGP training?

-	Have there been any changes in staff behaviour? Or staff management?

-	What would make this venue culture safer? What could be done?

11. If we wanted to see changes in NT music venues (less harms) what do you think needs to be done?

-	*What do you think are the barriers to changing venue culture ‘to creating a culture of care where people look out for each other and step in if trouble is kicking off’?*

*12.* Conclusion of interview

*Any other experiences or issues participant wants to raise that were not covered in the interview?*

Thank you so much for participating.

We appreciate your time and willingness to chat. A grocery voucher will be provided to you (physically, or electronically via text or email) after this interview as a thank you for participating.
