# Supplementary material for: Exploring the role of commercial actors and structures in contributing to sexual violence in live music venues
Source: Health Promot Int. 2026 Oct 6;41(5):daag148. doi: 10.1093/heapro/daag148 (PMC13639480; doi:10.1093/heapro/daag148)
Supplement: Supplementary file 1 [file daag148_supplementary_data.zip › Supplementary file 2 - Interview guide (patrons).docx]

**Supplementary File 2: Interview guide (patrons)**

Hi I am [name] from Menzies.

We are doing research with Music NT to help pubs and clubs respond better when there is trouble at venues.

We can have a 10-20 min chat to hear your thoughts.

At the end of the interview, we have a $40 woollies voucher to give you.

Its anonymous and confidential.

We can do the interview now or take your details and do it by phone or zoom at another time that works for you.

1.	Informed consent/plain language statement process.

2.	Information regarding participant

*•How often do you come to [venue name]*

*•Gender and pronouns*

*• Age*

3.	So, what I would like to begin with is with exploring what it is like in [VENUE NAME]

*How safe do you feel coming here?*

*Do you think its safe here for everyone?*

4.	Can you tell me about the venue culture at [venue name]?

-	What is it like to party here?

-	What do you think the reputation of the venue is for patrons?

*(Especially as it relates to the harms we talked about previously)*

- *Have there been any changes in the venue culture recently or over the last few years?*
- *Have there been any changes in staff behaviour?*
- *Does it matter to you what a venue culture is around this stuff? Would it make a difference to where you go? Do you think it matters to others?*
- *What would make this venue culture safer? What could be done?*

5.	Can you tell me about a time at [venue name] when you experienced behaviour that made you feel uncomfortable?

- *like someone being creepy, leering, following you around, rubbing up against you or groping you, catcalling you*
- *like someone harassing you,*
- *or aggressive or violent behaviour like pushing, slapping, punching, aggressive looks*
- *like being yelled at or someone being verbally abusive or derogatory or discriminatory,*
- *or like being discriminated against based on race, gender, disability, religion, or some other personal characteristic, experiencing racism*
- *or even more violent behaviour like assault*
- *What did the staff do?*
- *What did other people at [venue name] do?*
- *How often does that sort of thing happen?*
- *Do you hear about it happening to other people?*
- *Have there been other times for you?*

6.	Can you tell me about a time at [venue name] when you experienced behaviour that made you feel unsafe?

*-	What did the staff do?*

*-	What did other people at [venue name] do?*

*-	How often does that sort of thing happen?*

*-	Do you hear about it happening to other people?*

*-	Have there been other times for you?*

7. How would you expect staff at the venue to respond?

*Do you notice how performers respond? Or security? Or bar staff?*

8. Do you see other people stepping in to keep others safe?

*Could be friends or strangers*

9. All Good Project recall

- *Have you noticed any posters, materials or other messaging in this or other venues that relate to discrimination, harassment or violence or anything we’ve discussed so far?*
- *What have you seen/where did you see it?*
- *Have you seen this type of messaging in other places in the NT or elsewhere?*
- *Do you remember what you thought when you first saw it?*
- *This venue has signed up to a project called the All Good Project, which trains staff to respond to harassment and discrimination. Is there anything else you can recall that reflects this venue’s involvement?*
- *What do you think about venues being involved in these kinds of projects?*

10.	If we wanted to see changes in NT music venues (less harms) what do you think needs to be done?

*-	What do you think are the barriers to changing venue culture ‘to creating a culture of care, where people look out for each other and step in if trouble is kicking off ’?*

11.	Conclusion of interview

*Prompts include:*

*•	Any other experiences or issues participant wants to raise that were not covered in the interview?*

Thank you so much for participating.

We appreciate your time and willingness to chat. A grocery voucher will be provided to you (physically, or electronically via text or email) after this interview as a thank you for participating.
